# (*E*)-*N*-[(*E*)-3-(4-Nitro­phen­yl)allyl­idene]naphthalen-1-amine

**DOI:** 10.1107/S1600536813006417

**Published:** 2013-03-16

**Authors:** Kee Dal Nam, Joo Hwan Cha, Yong Seo Cho, Jae Kyun Lee, Ae Nim Pae

**Affiliations:** a Chemical Kinomics Research Center, Korea Institute of Science & Technology, Hwarangro 14-gil, Seongbuk-gu, Seoul 136-791, Republic of Korea; b Advanced Analysis Center, Korea Institute of Science & Technology, Hwarangro 14-gil, Seongbuk-gu, Seoul 136-791, Republic of Korea; c Center for Neuro-Medicine, Brain Science Institute, Korea Institute of Science & Technology, Hwarangro 14-gil, Seongbuk-gu, Seoul 136-791, Republic of Korea

## Abstract

In the title compound, C_19_H_14_N_2_O_2_, the dihedral angle between the mean planes of the 4-nitro­phenyl ring and the naphthalene ring system is 12.79 (2)°. The imine group displays a C—C—N=C torsion angle of 41.0 (2)° and the C=N group has an *E* conformation. In the crystal, weak C—H⋯O hydrogen bonds link molecules into layers parallel to the *b* axis.

## Related literature

For the synthesis and biological activity of naphthalene compounds, see: Upadhayaya *et al.* (2010 ▶); Rokade & Sayyed (2009 ▶). For a related structure, see: Yang *et al.* (2012 ▶).
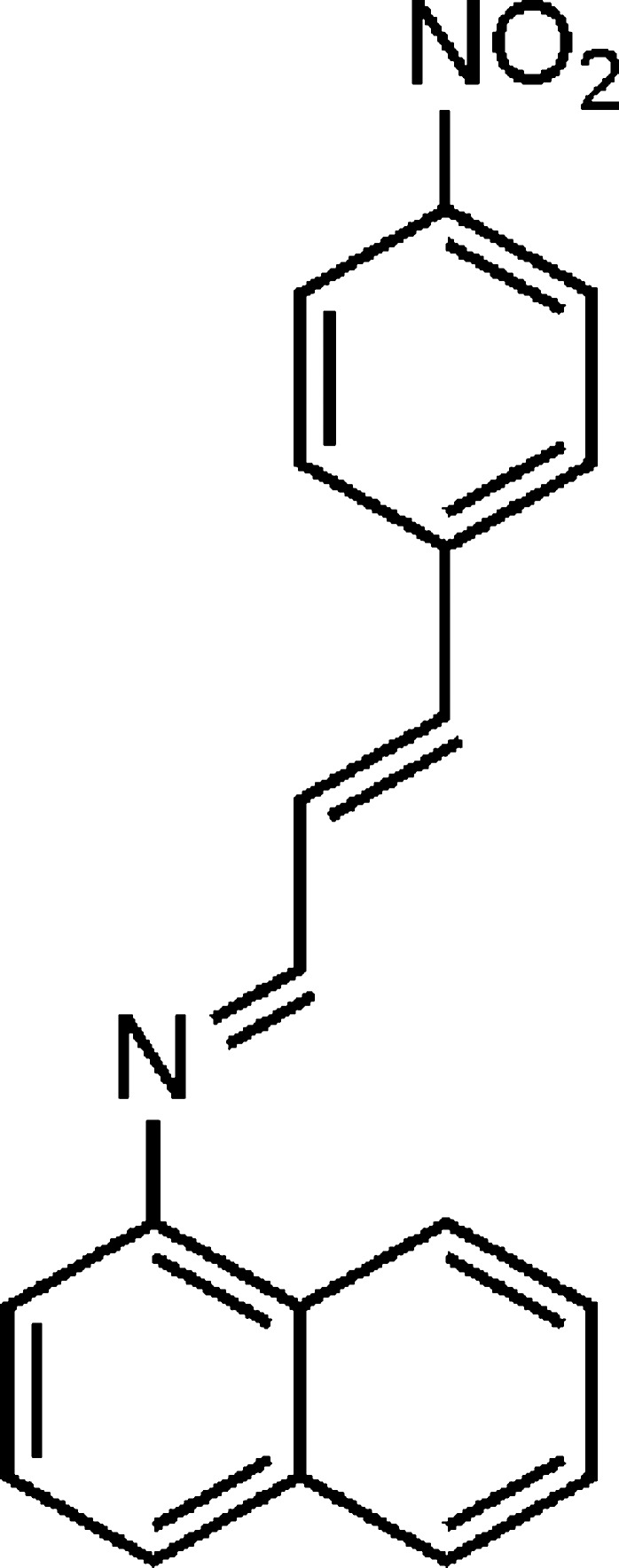


## Experimental

### 

#### Crystal data

C_19_H_14_N_2_O_2_
*M* *_r_* = 302.33
Monoclinic, 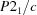

*a* = 7.7021 (5) Å
*b* = 13.8713 (12) Å
*c* = 14.2554 (10) Å
β = 98.096 (2)°
*V* = 1507.8 (2) Å^3^
*Z* = 4
Mo *K*α radiation
μ = 0.09 mm^−1^
*T* = 296 K
0.20 × 0.20 × 0.10 mm

#### Data collection

Rigaku R-AXIS RAPID diffractometer
Absorption correction: multi-scan (*ABSCOR*; Rigaku, 1995 ▶) *T*_min_ = 0.714, *T*_max_ = 0.991
14376 measured reflections
3426 independent reflections
1824 reflections with *F* ^2^ > 2σ(*F* ^2^)
*R*_int_ = 0.038

#### Refinement

*R*[*F* ^2^ > 2σ(*F* ^2^)] = 0.044
*wR*(*F* ^2^) = 0.125
*S* = 1.00
3426 reflections
220 parameters
H atoms treated by a mixture of independent and constrained refinement
Δρ_max_ = 0.16 e Å^−3^
Δρ_min_ = −0.24 e Å^−3^

### 

Data collection: *RAPID-AUTO* (Rigaku, 2006 ▶); cell refinement: *RAPID-AUTO*; data reduction: *RAPID-AUTO*; program(s) used to solve structure: *Il Milione* (Burla *et al.*, 2007 ▶); program(s) used to refine structure: *SHELXL97* (Sheldrick, 2008 ▶); molecular graphics: *CrystalStructure* (Rigaku, 2010 ▶); software used to prepare material for publication: *CrystalStructure*.

## Supplementary Material

Crystal structure: contains datablock(s) global, I. DOI: 10.1107/S1600536813006417/ff2100sup1.cif

Structure factors: contains datablock(s) I. DOI: 10.1107/S1600536813006417/ff2100Isup2.hkl

Supplementary material file. DOI: 10.1107/S1600536813006417/ff2100Isup3.cml

Additional supplementary materials:  crystallographic information; 3D view; checkCIF report

## Figures and Tables

**Table 1:** Hydrogen-bond geometry (Å, °)

*D*—H⋯*A*	*D*—H	H⋯*A*	*D*⋯*A*	*D*—H⋯*A*
C5—H5⋯O1^i^	0.93	2.66	3.422 (3)	139
C15—H15⋯O2^ii^	0.93	2.46	3.326 (3)	155

Symmetry codes: (i) 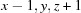; (ii) 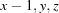.

**Fig. 1.**
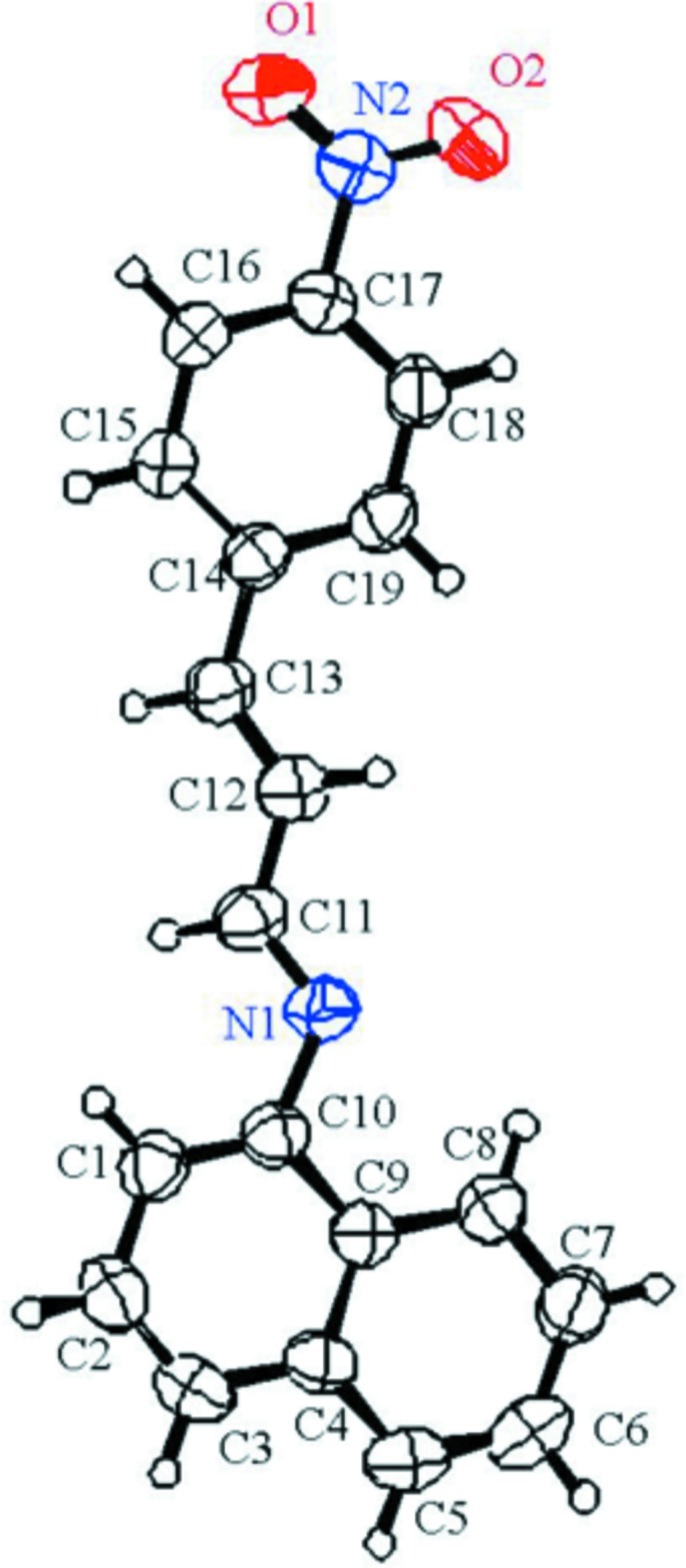
The molecular structure of the title compound showing the atomic numbering and 50% probability displacement ellipsoid.

## supplementary crystallographic information

### Comment

Naphthalene derivatives has been identified as new range of potent antimicrobials effective against wide range of human pathogens. They occupy a central place among medicinally important compounds due to their diverse and interesting antibiotic properties with minimum toxicity. (Rokade & Sayyed, 2009; Upadhayaya *et al.* 2010). In this paper, the title compound was synthesized and characterized by X-ray diffraction. Crystal structure of a similar compound, *N*-(Naphthalen-1-yl&shy;methyl&shy;idene)-4*H*-1,2,4-triazol-4-amine, was described previously by Yang *et al.* (2012).

In the title compound (Fig. 1), C_19_H_14_N_2_O_2_, the dihedral angle between the mean planes of the 4-nitrophenyl ring (C14—C19) and the naphthalene ring (C1—C10) is 12.79 (2)°. The imine group displays a torsion angle [C1–C10–N1–C11 = 41.0 (2)°] and the imine [C11 = N1] group has an (*E*) configuration. In the crystal, weak intermolecular C—H···O hydrogen bonds link molecules into layers parallel to the *b* axis.

### Experimental

To a solution of 1-naphthylamine (2.0 mmol) in anhydrous ethanol (40 ml) was treated with equimolar quantities of substituted 4-nitrocinnamaldehydes. The mixture was refluxed for 3 days, and the progress of reaction was monitored by TLC. After completion of reaction, the solvent was removed under reduced pressure. The residue was purified by flash column chromatography to afford the title compound as a yellow solid in yield 88%. Single crystals suitable for X-ray diffraction were prepared by slow evaporation of a solution of the title compound in ethanol at room temperature.

### Refinement

All hydrogen atoms were positioned geometrically and refined using a riding model with C—H = 0.93–0.98 Å and *U*iso(H) = 1.2 or 1.5 *U*eq(C).

### Crystal data

**Table:**

C_19_H_14_N_2_O_2_	*F*(000) = 632.00
*M**_r_* = 302.33	*D*_x_ = 1.332 Mg m^−^^3^
Monoclinic, *P*2_1_/*c*	Mo *K*α radiation, λ = 0.71075 Å
Hall symbol: -P 2ybc	Cell parameters from 7945 reflections
*a* = 7.7021 (5) Å	θ = 3.1–27.5°
*b* = 13.8713 (12) Å	µ = 0.09 mm^−^^1^
*c* = 14.2554 (10) Å	*T* = 296 K
β = 98.096 (2)°	Chunk, yellow
*V* = 1507.8 (2) Å^3^	0.20 × 0.20 × 0.10 mm
*Z* = 4	

### Data collection

**Table:**

Rigaku R-AXIS RAPID diffractometer	1824 reflections with *F*^2^ > 2σ(*F*^2^)
Detector resolution: 10.000 pixels mm^-1^	*R*_int_ = 0.038
ω scans	θ_max_ = 27.5°
Absorption correction: multi-scan (*ABSCOR*; Rigaku, 1995)	*h* = −8→9
*T*_min_ = 0.714, *T*_max_ = 0.991	*k* = −18→17
14376 measured reflections	*l* = −18→18
3426 independent reflections	

### Refinement

**Table:**

Refinement on *F*^2^	Secondary atom site location: difference Fourier map
*R*[*F*^2^ > 2σ(*F*^2^)] = 0.044	Hydrogen site location: inferred from neighbouring sites
*wR*(*F*^2^) = 0.125	H atoms treated by a mixture of independent and constrained refinement
*S* = 1.00	*w* = 1/[σ^2^(*F*_o_^2^) + (0.0632*P*)^2^] where *P* = (*F*_o_^2^ + 2*F*_c_^2^)/3
3426 reflections	(Δ/σ)_max_ < 0.001
220 parameters	Δρ_max_ = 0.16 e Å^−^^3^
0 restraints	Δρ_min_ = −0.24 e Å^−^^3^
Primary atom site location: structure-invariant direct methods	

### Special details

**Table:**

Geometry. All e.s.d.'s (except the e.s.d. in the dihedral angle between two l.s. planes) are estimated using the full covariance matrix. The cell e.s.d.'s are taken into account individually in the estimation of e.s.d.'s in distances, angles and torsion angles; correlations between e.s.d.'s in cell parameters are only used when they are defined by crystal symmetry. An approximate (isotropic) treatment of cell e.s.d.'s is used for estimating e.s.d.'s involving l.s. planes.
Refinement. Refinement was performed using all reflections. The weighted *R*-factor (*wR*) and goodness of fit (*S*) are based on *F*^2^. *R*-factor (gt) are based on *F*. The threshold expression of *F*^2^ > 2.0 σ(*F*^2^) is used only for calculating *R*-factor (gt).

### Fractional atomic coordinates and isotropic or equivalent isotropic displacement parameters (Å^2^)

**Table:**

	*x*	*y*	*z*	*U*_iso_*/*U*_eq_	
O1	0.83231 (18)	0.69908 (11)	0.01889 (9)	0.0893 (5)	
O2	1.04049 (17)	0.66549 (11)	0.13076 (10)	0.0912 (5)	
N1	0.28674 (17)	0.58150 (9)	0.58185 (9)	0.0559 (4)	
N2	0.8861 (2)	0.68108 (10)	0.10197 (11)	0.0639 (4)	
C1	−0.0251 (2)	0.57846 (11)	0.60108 (11)	0.0571 (4)	
C2	−0.1560 (2)	0.57496 (12)	0.65987 (13)	0.0646 (5)	
C3	−0.1124 (3)	0.57509 (12)	0.75554 (13)	0.0650 (5)	
C4	0.0649 (2)	0.57721 (10)	0.79753 (11)	0.0531 (4)	
C5	0.1143 (3)	0.57601 (12)	0.89707 (12)	0.0656 (5)	
C6	0.2852 (3)	0.57557 (13)	0.93612 (12)	0.0708 (5)	
C7	0.4176 (3)	0.57612 (12)	0.87819 (12)	0.0667 (5)	
C8	0.3764 (2)	0.57776 (11)	0.78197 (11)	0.0566 (4)	
C9	0.19908 (19)	0.57918 (10)	0.73900 (10)	0.0478 (4)	
C10	0.1495 (2)	0.58192 (10)	0.63815 (10)	0.0493 (4)	
C11	0.2713 (3)	0.63110 (12)	0.50566 (11)	0.0548 (4)	
C12	0.4042 (3)	0.62977 (12)	0.44420 (11)	0.0560 (4)	
C13	0.3893 (3)	0.67568 (12)	0.36151 (11)	0.0548 (4)	
C14	0.5200 (2)	0.67695 (10)	0.29619 (10)	0.0486 (4)	
C15	0.4674 (2)	0.69629 (11)	0.20060 (10)	0.0562 (4)	
C16	0.5861 (2)	0.69706 (11)	0.13708 (11)	0.0546 (4)	
C17	0.75962 (19)	0.67906 (10)	0.16958 (10)	0.0494 (4)	
C18	0.8178 (2)	0.66060 (11)	0.26364 (11)	0.0571 (5)	
C19	0.6975 (2)	0.65988 (11)	0.32628 (11)	0.0562 (4)	
H1	−0.0568	0.5784	0.5357	0.0685*	
H2	−0.2733	0.5725	0.6333	0.0775*	
H3	−0.2005	0.5738	0.7939	0.0780*	
H5	0.0277	0.5755	0.9364	0.0787*	
H6	0.3146	0.5749	1.0017	0.0850*	
H7	0.5346	0.5754	0.9055	0.0801*	
H8	0.4655	0.5779	0.7442	0.0679*	
H15	0.3498	0.7089	0.1793	0.0674*	
H16	0.5497	0.7095	0.0732	0.0655*	
H18	0.9359	0.6489	0.2843	0.0686*	
H19	0.7353	0.6478	0.3901	0.0674*	
H13	0.284 (3)	0.7118 (12)	0.3417 (11)	0.064 (5)*	
H12	0.507 (3)	0.5928 (12)	0.4638 (12)	0.072 (6)*	
H11	0.169 (3)	0.6735 (11)	0.4877 (11)	0.062 (5)*	

### Atomic displacement parameters (Å^2^)

**Table:**

	*U*^11^	*U*^22^	*U*^33^	*U*^12^	*U*^13^	*U*^23^
O1	0.0896 (10)	0.1221 (13)	0.0611 (8)	0.0165 (8)	0.0280 (8)	0.0112 (8)
O2	0.0506 (8)	0.1242 (12)	0.1018 (11)	0.0034 (8)	0.0215 (8)	0.0112 (9)
N1	0.0591 (8)	0.0612 (9)	0.0492 (8)	0.0021 (7)	0.0141 (7)	−0.0007 (7)
N2	0.0625 (10)	0.0652 (10)	0.0666 (10)	−0.0003 (8)	0.0182 (8)	0.0005 (8)
C1	0.0591 (10)	0.0585 (10)	0.0523 (9)	0.0027 (8)	0.0032 (8)	−0.0049 (8)
C2	0.0506 (10)	0.0699 (12)	0.0729 (12)	0.0021 (9)	0.0070 (9)	−0.0074 (9)
C3	0.0546 (10)	0.0716 (12)	0.0732 (12)	−0.0002 (9)	0.0235 (9)	−0.0052 (9)
C4	0.0587 (10)	0.0492 (9)	0.0541 (10)	0.0008 (7)	0.0171 (8)	−0.0025 (7)
C5	0.0788 (13)	0.0671 (11)	0.0552 (10)	−0.0008 (10)	0.0247 (10)	−0.0015 (9)
C6	0.0938 (15)	0.0728 (12)	0.0456 (9)	−0.0023 (11)	0.0087 (10)	0.0031 (8)
C7	0.0653 (11)	0.0727 (12)	0.0591 (10)	−0.0018 (9)	−0.0020 (9)	0.0068 (9)
C8	0.0553 (10)	0.0576 (10)	0.0572 (10)	0.0014 (8)	0.0094 (8)	0.0050 (8)
C9	0.0510 (9)	0.0427 (8)	0.0506 (9)	0.0012 (7)	0.0099 (8)	0.0001 (7)
C10	0.0545 (9)	0.0465 (9)	0.0483 (9)	0.0029 (7)	0.0116 (8)	−0.0019 (7)
C11	0.0607 (10)	0.0556 (10)	0.0487 (9)	0.0012 (9)	0.0103 (8)	−0.0024 (8)
C12	0.0608 (11)	0.0573 (10)	0.0510 (10)	0.0006 (9)	0.0116 (9)	−0.0004 (8)
C13	0.0578 (10)	0.0553 (10)	0.0518 (10)	0.0021 (8)	0.0096 (8)	0.0003 (8)
C14	0.0532 (9)	0.0456 (9)	0.0469 (9)	0.0002 (7)	0.0064 (7)	0.0010 (7)
C15	0.0486 (9)	0.0686 (11)	0.0504 (9)	0.0068 (8)	0.0040 (8)	0.0046 (8)
C16	0.0551 (10)	0.0633 (10)	0.0444 (9)	0.0030 (8)	0.0039 (8)	0.0037 (8)
C17	0.0494 (9)	0.0478 (9)	0.0525 (9)	−0.0008 (7)	0.0121 (8)	0.0011 (7)
C18	0.0479 (9)	0.0620 (11)	0.0594 (10)	0.0011 (8)	0.0003 (8)	0.0054 (8)
C19	0.0580 (10)	0.0645 (11)	0.0437 (9)	0.0002 (8)	−0.0014 (8)	0.0050 (8)

### Geometric parameters (Å, º)

**Table:**

O1—N2	1.224 (2)	C14—C19	1.395 (3)
O2—N2	1.222 (2)	C15—C16	1.375 (3)
N1—C10	1.414 (3)	C16—C17	1.374 (2)
N1—C11	1.277 (2)	C17—C18	1.377 (2)
N2—C17	1.464 (3)	C18—C19	1.374 (3)
C1—C2	1.400 (3)	C1—H1	0.930
C1—C10	1.375 (2)	C2—H2	0.930
C2—C3	1.358 (3)	C3—H3	0.930
C3—C4	1.412 (3)	C5—H5	0.930
C4—C5	1.416 (3)	C6—H6	0.930
C4—C9	1.417 (3)	C7—H7	0.930
C5—C6	1.355 (3)	C8—H8	0.930
C6—C7	1.400 (3)	C11—H11	0.989 (16)
C7—C8	1.364 (3)	C12—H12	0.953 (17)
C8—C9	1.416 (2)	C13—H13	0.959 (17)
C9—C10	1.435 (2)	C15—H15	0.930
C11—C12	1.438 (3)	C16—H16	0.930
C12—C13	1.330 (3)	C18—H18	0.930
C13—C14	1.464 (3)	C19—H19	0.930
C14—C15	1.392 (2)		
			
O1···C16	2.708 (3)	C7···H13^viii^	3.134 (17)
O1···C18	3.547 (2)	C8···H3^v^	3.2394
O2···C16	3.540 (2)	C8···H15^viii^	3.2947
O2···C18	2.729 (3)	C8···H13^viii^	3.148 (17)
N1···C8	2.840 (2)	C9···H15^viii^	3.3151
N1···C13	3.591 (3)	C9···H18^iv^	3.3321
C1···C4	2.789 (3)	C9···H13^viii^	3.272 (16)
C1···C11	2.910 (3)	C10···H1^xi^	3.3316
C2···C9	2.808 (2)	C10···H15^viii^	3.2996
C3···C10	2.798 (3)	C10···H18^iv^	3.4799
C4···C7	2.797 (3)	C10···H19^iv^	3.3466
C5···C8	2.775 (3)	C11···H1^xi^	3.3541
C6···C9	2.795 (3)	C11···H2^xi^	3.4512
C9···C11	3.523 (3)	C11···H15^viii^	3.3164
C12···C19	3.029 (3)	C11···H16^viii^	3.1354
C14···C17	2.758 (3)	C11···H12^iv^	3.540 (17)
C15···C18	2.767 (3)	C12···H2^v^	3.4901
C16···C19	2.761 (3)	C12···H2^xi^	3.1301
O1···C1^i^	3.426 (3)	C12···H16^viii^	3.0046
O1···C5^ii^	3.422 (3)	C12···H12^iv^	3.386 (17)
O1···C19^iii^	3.412 (2)	C13···H2^xi^	3.5604
O2···C5^iv^	3.559 (3)	C13···H16^viii^	3.4816
O2···C15^v^	3.326 (2)	C13···H18^ix^	3.5298
N1···C19^iv^	3.591 (2)	C14···H8^iv^	3.5866
N2···C1^i^	3.405 (2)	C14···H8^iii^	3.4924
N2···C2^i^	3.509 (3)	C15···H6^x^	3.3647
N2···C5^iv^	3.566 (3)	C15···H8^iii^	3.1933
C1···O1^vi^	3.426 (3)	C16···H2^i^	3.3773
C1···N2^vi^	3.405 (2)	C16···H6^x^	3.1359
C2···N2^vi^	3.509 (3)	C17···H2^i^	3.4883
C2···C17^vi^	3.480 (3)	C19···H1^v^	3.4892
C4···C18^iv^	3.561 (3)	H1···O1^vi^	3.2020
C5···O1^vii^	3.422 (3)	H1···N1^xi^	3.1668
C5···O2^iv^	3.559 (3)	H1···N2^vi^	3.5116
C5···N2^iv^	3.566 (3)	H1···C1^xi^	3.0465
C7···C13^viii^	3.456 (3)	H1···C10^xi^	3.3316
C8···C14^viii^	3.575 (2)	H1···C11^xi^	3.3541
C8···C15^viii^	3.448 (3)	H1···C19^ix^	3.4892
C9···C18^iv^	3.329 (2)	H1···H1^xi^	2.6052
C9···C19^iv^	3.564 (3)	H1···H19^ix^	2.6192
C10···C19^iv^	3.567 (2)	H1···H12^ix^	3.3760
C13···C7^iii^	3.456 (3)	H2···N1^ix^	3.3669
C14···C8^iii^	3.575 (2)	H2···C11^xi^	3.4512
C15···O2^ix^	3.326 (2)	H2···C12^ix^	3.4901
C15···C8^iii^	3.448 (3)	H2···C12^xi^	3.1301
C17···C2^i^	3.480 (3)	H2···C13^xi^	3.5604
C18···C4^iv^	3.561 (3)	H2···C16^vi^	3.3773
C18···C9^iv^	3.329 (2)	H2···C17^vi^	3.4883
C19···O1^viii^	3.412 (2)	H2···H8^ix^	2.7294
C19···N1^iv^	3.591 (2)	H2···H16^vi^	3.3765
C19···C9^iv^	3.564 (3)	H2···H12^ix^	2.7617
C19···C10^iv^	3.567 (2)	H2···H12^xi^	3.1167
O1···H16	2.4154	H3···C7^ix^	3.3298
O2···H18	2.4477	H3···C8^ix^	3.2394
N1···H1	2.6358	H3···H7^ix^	2.7580
N1···H8	2.5219	H3···H8^ix^	2.5703
N1···H12	2.558 (19)	H5···O1^vii^	2.6614
N2···H16	2.5955	H5···O2^vii^	3.0276
N2···H18	2.6122	H5···O2^iv^	3.4966
C1···H3	3.2302	H5···N2^vii^	3.1031
C1···H11	2.695 (17)	H5···C5^xii^	3.4610
C3···H1	3.2230	H5···H5^xii^	2.8416
C3···H5	2.6512	H5···H6^xii^	3.5690
C4···H2	3.2486	H6···O2^vii^	3.2431
C4···H6	3.2546	H6···C7^xiii^	3.2526
C4···H8	3.2801	H6···C15^xiv^	3.3647
C5···H3	2.6530	H6···C16^xiv^	3.1359
C5···H7	3.2228	H6···H5^xii^	3.5690
C6···H8	3.2377	H6···H6^xiii^	3.5374
C7···H5	3.2269	H6···H7^xiii^	2.6476
C8···H6	3.2337	H6···H15^xiv^	3.1225
C9···H1	3.2677	H6···H16^xiv^	2.6995
C9···H3	3.2819	H7···O1^xiv^	3.1257
C9···H5	3.2729	H7···C6^xiii^	3.2438
C9···H7	3.2542	H7···H3^v^	2.7580
C10···H2	3.2503	H7···H6^xiii^	2.6476
C10···H8	2.6791	H7···H7^xiii^	3.5092
C10···H11	2.515 (16)	H7···H16^xiv^	3.0192
C11···H1	2.7219	H7···H13^viii^	3.5723
C11···H8	3.5939	H8···C2^v^	3.3067
C11···H13	2.607 (16)	H8···C3^v^	3.2321
C12···H19	2.7779	H8···C14^iv^	3.5866
C13···H15	2.6129	H8···C14^viii^	3.4924
C13···H19	2.6670	H8···C15^viii^	3.1933
C13···H11	2.642 (17)	H8···H2^v^	2.7294
C14···H16	3.2501	H8···H3^v^	2.5703
C14···H18	3.2552	H8···H15^viii^	3.1875
C14···H12	2.673 (17)	H8···H13^viii^	3.5949
C15···H19	3.2306	H15···O2^ix^	2.4604
C15···H13	2.622 (17)	H15···N1^iii^	3.2294
C16···H18	3.2430	H15···C8^iii^	3.2947
C17···H15	3.2058	H15···C9^iii^	3.3151
C17···H19	3.2044	H15···C10^iii^	3.2996
C18···H16	3.2425	H15···C11^iii^	3.3164
C19···H15	3.2315	H15···H6^x^	3.1225
C19···H13	3.300 (18)	H15···H8^iii^	3.1875
C19···H12	2.769 (18)	H15···H11^iii^	3.3154
H1···H2	2.3187	H16···N1^iii^	3.5481
H1···H11	2.3587	H16···C6^x^	3.2099
H2···H3	2.2795	H16···C7^x^	3.3756
H3···H5	2.4921	H16···C11^iii^	3.1354
H5···H6	2.2740	H16···C12^iii^	3.0046
H6···H7	2.3246	H16···C13^iii^	3.4816
H7···H8	2.2861	H16···H2^i^	3.3765
H15···H16	2.3051	H16···H6^x^	2.6995
H15···H13	2.4371	H16···H7^x^	3.0192
H18···H19	2.3055	H16···H12^iii^	3.1495
H19···H13	3.5569	H16···H11^iii^	3.4229
H19···H12	2.3008	H18···C1^iv^	3.5734
H13···H12	2.80 (3)	H18···C2^iv^	3.5731
H13···H11	2.44 (3)	H18···C3^iv^	3.4701
H12···H11	2.90 (3)	H18···C4^iv^	3.3461
O1···H1^i^	3.2020	H18···C9^iv^	3.3321
O1···H5^ii^	2.6614	H18···C10^iv^	3.4799
O1···H7^x^	3.1257	H18···C13^v^	3.5298
O1···H19^iii^	2.8387	H18···H13^v^	2.8315
O1···H11^i^	3.219 (17)	H18···H11^v^	3.2054
O2···H5^ii^	3.0276	H19···O1^viii^	2.8387
O2···H5^iv^	3.4966	H19···N1^iv^	3.2127
O2···H6^ii^	3.2431	H19···C1^v^	3.4374
O2···H15^v^	2.4604	H19···C10^iv^	3.3466
O2···H13^v^	3.375 (15)	H19···H1^v^	2.6192
O2···H11^i^	3.269 (16)	H19···H11^v^	3.4503
N1···H1^xi^	3.1668	H13···O2^ix^	3.375 (15)
N1···H2^v^	3.3669	H13···C4^iii^	3.394 (17)
N1···H15^viii^	3.2294	H13···C5^iii^	3.360 (17)
N1···H16^viii^	3.5481	H13···C6^iii^	3.241 (16)
N1···H19^iv^	3.2127	H13···C7^iii^	3.134 (17)
N1···H12^iv^	3.013 (17)	H13···C8^iii^	3.148 (17)
N2···H1^i^	3.5116	H13···C9^iii^	3.272 (16)
N2···H5^ii^	3.1031	H13···H7^iii^	3.5723
N2···H11^i^	3.530 (17)	H13···H8^iii^	3.5949
C1···H1^xi^	3.0465	H13···H18^ix^	2.8315
C1···H18^iv^	3.5734	H12···N1^iv^	3.013 (17)
C1···H19^ix^	3.4374	H12···C2^v^	3.545 (16)
C2···H8^ix^	3.3067	H12···C11^iv^	3.540 (17)
C2···H18^iv^	3.5731	H12···C12^iv^	3.386 (17)
C2···H12^ix^	3.545 (16)	H12···H1^v^	3.3760
C3···H8^ix^	3.2321	H12···H2^v^	2.7617
C3···H18^iv^	3.4701	H12···H2^xi^	3.1167
C4···H18^iv^	3.3461	H12···H16^viii^	3.1495
C4···H13^viii^	3.394 (17)	H12···H12^iv^	2.78 (3)
C5···H5^xii^	3.4610	H11···O1^vi^	3.219 (17)
C5···H13^viii^	3.360 (17)	H11···O2^vi^	3.269 (16)
C6···H7^xiii^	3.2438	H11···N2^vi^	3.530 (17)
C6···H16^xiv^	3.2099	H11···H15^viii^	3.3154
C6···H13^viii^	3.241 (16)	H11···H16^viii^	3.4229
C7···H3^v^	3.3298	H11···H18^ix^	3.2054
C7···H6^xiii^	3.2526	H11···H19^ix^	3.4503
C7···H16^xiv^	3.3756		
			
C10—N1—C11	119.53 (14)	C17—C18—C19	118.51 (15)
O1—N2—O2	122.81 (17)	C14—C19—C18	121.37 (14)
O1—N2—C17	118.37 (14)	C2—C1—H1	119.358
O2—N2—C17	118.82 (15)	C10—C1—H1	119.366
C2—C1—C10	121.28 (15)	C1—C2—H2	119.856
C1—C2—C3	120.30 (15)	C3—C2—H2	119.847
C2—C3—C4	120.87 (17)	C2—C3—H3	119.562
C3—C4—C5	122.11 (17)	C4—C3—H3	119.566
C3—C4—C9	119.53 (15)	C4—C5—H5	119.342
C5—C4—C9	118.36 (15)	C6—C5—H5	119.342
C4—C5—C6	121.32 (18)	C5—C6—H6	119.866
C5—C6—C7	120.27 (16)	C7—C6—H6	119.866
C6—C7—C8	120.49 (16)	C6—C7—H7	119.748
C7—C8—C9	120.61 (16)	C8—C7—H7	119.758
C4—C9—C8	118.94 (14)	C7—C8—H8	119.691
C4—C9—C10	118.51 (13)	C9—C8—H8	119.700
C8—C9—C10	122.54 (15)	N1—C11—H11	121.1 (10)
N1—C10—C1	123.40 (13)	C12—C11—H11	117.4 (10)
N1—C10—C9	116.99 (13)	C11—C12—H12	117.6 (11)
C1—C10—C9	119.47 (15)	C13—C12—H12	118.8 (11)
N1—C11—C12	121.48 (16)	C12—C13—H13	118.4 (10)
C11—C12—C13	123.62 (16)	C14—C13—H13	115.6 (10)
C12—C13—C14	126.01 (16)	C14—C15—H15	119.424
C13—C14—C15	119.57 (14)	C16—C15—H15	119.428
C13—C14—C19	122.30 (14)	C15—C16—H16	120.586
C15—C14—C19	118.13 (15)	C17—C16—H16	120.584
C14—C15—C16	121.15 (14)	C17—C18—H18	120.741
C15—C16—C17	118.83 (14)	C19—C18—H18	120.752
N2—C17—C16	118.69 (13)	C14—C19—H19	119.312
N2—C17—C18	119.29 (14)	C18—C19—H19	119.319
C16—C17—C18	122.01 (15)		
			
C10—N1—C11—C12	−177.42 (12)	C6—C7—C8—C9	0.2 (3)
C11—N1—C10—C1	41.0 (2)	C7—C8—C9—C4	−1.1 (2)
C11—N1—C10—C9	−143.39 (13)	C7—C8—C9—C10	179.35 (13)
O1—N2—C17—C16	0.2 (2)	C4—C9—C10—N1	−178.53 (11)
O1—N2—C17—C18	−178.89 (13)	C4—C9—C10—C1	−2.72 (19)
O2—N2—C17—C16	179.70 (13)	C8—C9—C10—N1	1.1 (2)
O2—N2—C17—C18	0.6 (2)	C8—C9—C10—C1	176.87 (12)
C2—C1—C10—N1	177.29 (13)	N1—C11—C12—C13	175.92 (14)
C2—C1—C10—C9	1.8 (3)	C11—C12—C13—C14	179.95 (14)
C10—C1—C2—C3	0.1 (3)	C12—C13—C14—C15	156.74 (15)
C1—C2—C3—C4	−1.0 (3)	C12—C13—C14—C19	−23.6 (3)
C2—C3—C4—C5	−179.08 (14)	C13—C14—C15—C16	−179.30 (13)
C2—C3—C4—C9	−0.0 (3)	C13—C14—C19—C18	179.44 (13)
C3—C4—C5—C6	178.28 (14)	C15—C14—C19—C18	−0.9 (2)
C3—C4—C9—C8	−177.73 (12)	C19—C14—C15—C16	1.1 (2)
C3—C4—C9—C10	1.9 (2)	C14—C15—C16—C17	−0.5 (3)
C5—C4—C9—C8	1.4 (2)	C15—C16—C17—N2	−179.27 (12)
C5—C4—C9—C10	−179.04 (12)	C15—C16—C17—C18	−0.2 (3)
C9—C4—C5—C6	−0.8 (3)	N2—C17—C18—C19	179.39 (12)
C4—C5—C6—C7	−0.1 (3)	C16—C17—C18—C19	0.3 (3)
C5—C6—C7—C8	0.4 (3)	C17—C18—C19—C14	0.3 (3)

Symmetry codes: (i) *x*+1, −*y*+3/2, *z*−1/2; (ii) *x*+1, *y*, *z*−1; (iii) *x*, −*y*+3/2, *z*−1/2; (iv) −*x*+1, −*y*+1, −*z*+1; (v) *x*+1, *y*, *z*; (vi) *x*−1, −*y*+3/2, *z*+1/2; (vii) *x*−1, *y*, *z*+1; (viii) *x*, −*y*+3/2, *z*+1/2; (ix) *x*−1, *y*, *z*; (x) *x*, *y*, *z*−1; (xi) −*x*, −*y*+1, −*z*+1; (xii) −*x*, −*y*+1, −*z*+2; (xiii) −*x*+1, −*y*+1, −*z*+2; (xiv) *x*, *y*, *z*+1.

### Hydrogen-bond geometry (Å, º)

**Table:**

*D*—H···*A*	*D*—H	H···*A*	*D*···*A*	*D*—H···*A*
C5—H5···O1^vii^	0.93	2.66	3.422 (3)	139
C15—H15···O2^ix^	0.93	2.46	3.326 (3)	155

Symmetry codes: (vii) *x*−1, *y*, *z*+1; (ix) *x*−1, *y*, *z*.
